# Differential Activation of Glioprotective Intracellular Signaling Pathways in Primary Optic Nerve Head Astrocytes after Treatment with Different Classes of Antioxidants

**DOI:** 10.3390/antiox9040324

**Published:** 2020-04-16

**Authors:** Anita K. Ghosh, Vidhya R. Rao, Victoria J. Wisniewski, Alexandra D. Zigrossi, Jamie Floss, Peter Koulen, Evan B Stubbs, Simon Kaja

**Affiliations:** 1 Graduate Program in Biochemistry and Molecular Biology, Loyola University Chicago, Health Sciences Campus, Maywood, IL 60153, USA; 2 Research Service, Edward Hines Jr. Veterans Administration Hospital, Hines, IL 60141, USA; 3 Department of Ophthalmology, Loyola University Chicago, Stritch School of Medicine, Maywood, IL 60153, USA; 4 Department of Molecular Pharmacology and Neuroscience, Loyola University Chicago, Stritch School of Medicine, Maywood, IL 60153, USA; 5 Department of Ophthalmology and Biomedical Sciences, Vision Research Center, University of Missouri—Kansas City, School of Medicine, Vision Research Center, Kansas City, MO 64108, USA

**Keywords:** glioprotection, xanthohumol, resveratrol, manganese porphyrin, optic nerve head astrocytes, phase II antioxidant enzymes, cell viability

## Abstract

Optic nerve head astrocytes are the specialized glia cells that provide structural and trophic support to the optic nerve head. In response to cellular injury, optic nerve head astrocytes undergo reactive astrocytosis, the process of cellular activation associated with cytoskeletal remodeling, increases in the rate of proliferation and motility, and the generation of Reactive Oxygen Species. Antioxidant intervention has previously been proposed as a therapeutic approach for glaucomatous optic neuropathy, however, little is known regarding the response of optic nerve head astrocytes to antioxidants under physiological versus pathological conditions. The goal of this study was to determine the effects of three different antioxidants, manganese (III) tetrakis (1-methyl-4-pyridyl) porphyrin (Mn-TM-2-PyP), resveratrol and xanthohumol in primary optic nerve head astrocytes. Effects on the expression of the master regulator nuclear factor erythroid 2-related factor 2 (Nrf2), the antioxidant enzyme, manganese-dependent superoxide dismutase 2 (SOD2), and the pro-oxidant enzyme, nicotinamide adenine dinucleotide phosphate oxidase 4 (NOX4), were determined by quantitative immunoblotting. Furthermore, efficacy in preventing chemically and reactive astrocytosis-induced increases in cellular oxidative stress was quantified using cell viability assays. The results were compared to the effects of the prototypic antioxidant, Trolox. Antioxidants elicited highly differential changes in the expression levels of Nrf2, SOD2, and NOX4. Notably, Mn-TM-2-PyP increased SOD2 expression eight-fold, while resveratrol increased Nrf2 expression three-fold. In contrast, xanthohumol exerted no statistically significant changes in expression levels. 3-(4,5-dimethylthiazol-2-yl)-2,5-diphenyltetrazolium bromide (MTT) uptake and lactate dehydrogenase (LDH) release assays were performed to assess cell viability after chemically and reactive astrocytosis-induced oxidative stress. Mn-TM-2-PyP exerted the most potent glioprotection by fully preventing the loss of cell viability, whereas resveratrol and xanthohumol partially restored cell viability. Our data provide the first evidence for a well-developed antioxidant defense system in optic nerve head astrocytes, which can be pharmacologically targeted by different classes of antioxidants.

## 1. Introduction

Optic nerve head astrocytes are the specialized glia cells that provide structural and trophic support to the unmyelinated optic nerve head. The optic nerve head is the point of exit where axons of retinal ganglion cells leave the eye to form the axons of the optic nerve. Over the past few decades, complex signaling pathways have been identified in astrocytes that were previously thought to be restricted to neurons, including complex calcium signaling pathways [1]. Knowledge of the specific intracellular signaling pathways in optic nerve head astrocytes, however, remains limited and, due to the unique biomechanical environment of the optic nerve head, the translatability of findings from other types of astrocytes is limited.

In response to cellular triggers that can include biomechanical, bioenergetic and biochemical changes, optic nerve head astrocytes undergo reactive astrocytosis (also commonly referred to as astrogliosis) [2,3,4]. This cellular activation is associated with cytoskeletal remodeling, increases in the rate of proliferation and motility, and generation of Reactive Oxygen Species.

Previous studies by us and others have shown that primary optic nerve head astrocytes are vulnerable to the deleterious effects of oxidative stress and that antioxidants can protect against chemically induced oxidative stress [5,6,7,8]. Investigations into the underlying mechanisms, however, have primarily focused on the expression of apoptosis-related genes and proteins, such as caspases, Bcl-2, Bcl-2-associated protein and related signaling pathways [9,10].

The Kelch-like ECH-associated protein 1/nuclear factor erythroid 2 related factor 2/antioxidant response element (Keap1/Nrf2/ARE) pathway is the master regulator of phase II antioxidant enzymes, a group of enzymes critical for the endogenous antioxidant response (for review, see [11]). Pharmaceutical modulation of this pathway is of significant interest for the treatment of neurological, neurodegenerative and neuroimmunological disorders.

Therefore, the goal of the present study was to determine whether different classes of antioxidants can elicit selective responses in the endogenous antioxidant system and whether they exert glioprotective effects in optic nerve head astrocytes against both chemically induced and reactive astrocytosis-associated increases in oxidative stress.

For this study, three different antioxidants were evaluated: manganese (III) tetrakis (1-methyl-4-pyridyl) porphyrin (Mn-TM-2-PyP), resveratrol, and xanthohumol. The effects of these antioxidants were compared to the prototypic antioxidant, 6-hydroxy-2,5,7,8-tetramethylchroman-2-carboxylic acid (Trolox).

Mn-TM-2-PyP is a superoxide dismutase (SOD) mimetic that belongs to the metalloporphyrin group and possesses broad antioxidant specificity, which includes scavenging O_2_·^–^, H_2_O_2_, ONOO^-^, NO·, and lipid peroxyl radicals [12]. Manganese porphyrins are well characterized and offer protection in a variety of oxidative stress injuries such as stroke, diabetes, radiation injury, dry eye disease and ischemia [13,14,15,16,17,18,19].

Resveratrol is a stilbenoid polyphenol, well-known for its abundance in grape skin, where it is produced under injurious conditions [20]. There is controversy over the specific molecular mechanism of action of resveratrol. Data suggest several distinct pathways contributing to the tissue-specific effects of resveratrol, including its direct Reactive Oxygen Species (ROS) scavenging activity, the modulation of the endogenous antioxidant system through Nrf2 upregulation [21,22], and control over apoptosis-related genes.

Xanthohumol is a naturally occurring prenylated chalconoid abundantly present in *Humulus lupulus*, the hops plant. Xanthohumol exerts its antioxidant effects by stimulating the dissociation of Keap1 from Nrf2, allowing Nrf2 to translocate into the nucleus and bind the ARE, ultimately promoting the transcription of phase II antioxidant enzymes [23].

Lastly, Trolox is a water-soluble analog of vitamin E and ROS scavenger. Trolox is the most commonly used reference standard for the antioxidant capacity of compounds [24], partly due to its lack of modulation of intracellular antioxidant enzymes [25].

## 2. Materials and Methods

### 2.1. Antioxidants and Antibodies

Resveratrol and xanthohumol were purchased from Cayman Chemicals (Ann Arbor, MI, USA). Trolox and Mn-TM-2-PyP were obtained from Millipore Sigma (St. Louis, MO, USA). For experiments, resveratrol and xanthohumol were dissolved in dimethyl sulfoxide (Millipore Sigma, St. Louis, MO, USA), Mn-TM-2-PyP was dissolved in 0.1 M phosphate-buffered saline pH 7.4 (PBS) and Trolox was dissolved in ethanol.

The following antibodies were used for immunoblotting experiments: chicken anti-glial fibrillary acid protein (GFAP; ab4674; AbCam, Cambridge, MA, USA; 1:5000 dilution); rabbit anti-Nox4 (ab133303; AbCam; 1:5000 dilution); mouse anti-Nrf2 (VMA00224; Biorad Laboratories, Hercules, CA; 1:1000 dilution); rabbit anti-Sod2 (A1340; ABclonal, Woburn, MA; 1:2000 dilution). Glyceraldehyde-3-phosphate dehydrogenase (GAPDH) was used as endogenous control (rabbit anti-GAPDH; sc-25778; Santa Cruz Biotechnology, Dallas, TX; 1:2000 dilution). Secondary antibodies were horseradish peroxidase-conjugated and obtained from GE Healthcare (Chicago, IL, USA).

Immunocytochemistry was performed using chicken anti-glial fibrillary acid protein (GFAP; ab4674; AbCam; 1:1000 dilution) and Alexa Fluor^®^ 594-labeled goat anti-chicken secondary antibody (Thermo Fisher Scientific, Waltham, MA, USA).

### 2.2. Primary Culture of Optic Nerve Head Astrocytes

Primary cultures of adult rat optic nerve head astrocytes were maintained and validated as we have previously described in detail [5,6,26]. Cultures of passages 10 to 20 were used for experiments.

### 2.3. Induction of Reactive Astrocytosis

Optic nerve head astrocyte cultures were seeded at a density of 25,000 cells/cm^2^ in 6-well plates (TPP; Midwest Scientific, Valley Park, MO, USA). Cultures were exposed to 25-35 mm Hg pressure above ambient pressure for 16 h in a custom-built, vacuum-sealed hyperbaric pressure chamber placed inside of a 37 °C tissue culture incubator. Pressure was monitored through a liquid-immersed pressure sensor (Harvard Apparatus, Holliston, MA, USA), connected to a laptop computer running PowerLab 8/35 high-performance data acquisition system supplied with LabChart Pro software and modules (ADInstruments, Colorado Springs, CO, USA). Cultures were returned to ambient pressure for 1 h prior to experiments.

### 2.4. Protein Extraction and Immunoblotting

Media was aspirated and cells were washed and scraped in ice-cold PBS. Samples were centrifuged at 800 × g for 5 min; supernatant was aspirated, and pellets were lysed in five volumes of Cytobuster lysis reagent (Millipore Sigma St. Louis, MO, USA) supplemented with a protease inhibitor cocktail (Thermo Fisher Scientific, Waltham, MA, USA). Lysates were triturated with a 31-gauge insulin syringe and subsequently centrifuged at 16,000 × *g* for 10 min to remove cell debris. The protein concentrations of lysates were determined using the method of Lowry [27].

For immunoblotting, protein samples with loading buffer were denatured at 85 °C for 5 min. A total of 5–10 μg of each protein sample was loaded on pre-cast 4–12% NuPage^®^ Bis/Tris gels (Thermo Fisher Scientific, Waltham, MA, USA) and electrophoresed at 150 V for 75 min. Proteins were transferred from gels to nitrocellulose membrane with 0.2 μM pore size (Amersham Protran, GE Healthcare, Chicago, IL, USA) by wet-transfer using Pierce^TM^ Methanol-free Western Blot Transfer Buffer (Thermo Fisher Scientific, Waltham, MA, USA) at 100 V for 90 min. Membranes were blocked in 5% non-fat milk in PBS supplemented with 0.2% Tween-20 (PBS-T; Millipore Sigma, St. Louis, MO, USA), then incubated with primary antibody in 2.5% milk in PBS-T at 4 °C overnight while gently shaking. All primary antibodies used are listed in Section 2.1 with catalog numbers and dilutions; GAPDH was used as an endogenous control for all experiments. Membranes were washed three times in PBS-T then subsequently incubated with horseradish peroxidase-linked secondary antibody in 2.5% milk in PBS-T at room temperature for 1 h. Immunoblot detection was performed by chemiluminescence using the Luminata Forte^®^ reagent (Millipore Sigma, St. Louis, MO, USA). Images were acquired using a ChemiDoc^TM^ XRS+ System (Bio-Rad Laboratories, Hercules, CA, USA). Protein bands were analyzed by densitometry using either Image Lab software (Bio-Rad Laboratories, Hercules, CA, USA) or Fiji software (ImageJ, NIH, Bethesda, MD, USA), and normalized to GAPDH and the control condition.

### 2.5. Immunocytochemistry

Optic nerve head astrocytes were seeded on either black/clear bottom 96-well plates or 8-well chamber slides at a density of 25,000 cells/cm^2^. After two days, cells were rinsed in 100 μL PBS and subsequently fixed in 4% paraformaldehyde (PFA) in PBS for 15 min. Cultures were immunostained as described by us previously [26]. Cells were co-labeled with DAPI (NucBlue^TM^ Fixed Cell ReadyProbes^TM^ Reagent, Thermo Fisher Scientific, Waltham, MA, USA) to label cell nuclei. Images were acquired using a plate reader (Cytation5, Biotek, Winooski, VT, USA) or Leica SP5 confocal microscope (Leica Microsystems Inc., Buffalo Grove, IL, USA). Immunocytochemistry was analyzed by microfluorimetry using Fiji software, as described by our laboratory previously [28].

### 2.6. Filamentous and Globular Actin Staining

To label astrocyte cultures for filamentous (F-) and globular (G-) actin, cells were seeded on black/clear bottom 96-well plates or 8-well chamber slides at a density of 25,000 cells/cm^2^. After treatments, cells were rinsed in PBS then fixed in 4% PFA for 15 min. Cells were subsequently washed 3 × in PBS for 5 min each, then permeabilized in 0.1% Triton-X 100 for 5 min. Stain solution was prepared by adding two drops of ActinGreen^TM^ ReadyProbes^TM^ Reagent (Thermo Fisher Scientific, Waltham, MA, USA) for filamentous (F-) actin and two drops of NucBlue^TM^ Fixed Cell ReadyProbes^TM^ Reagent (Thermo Fisher Scientific, Waltham, MA, USA) to stain nuclei, per 1 mL of PBS. Alexa Fluor^TM^ 594 conjugated DNAseI (Thermo Fisher Scientific, Waltham, MA, USA) to stain globular (G-) actin was added to the stain solution for a final concentration of 0.3 μM, per the manufacturer’s instructions. A total of 100 μl stain solution was added to each well and incubated for 30 min at room temperature in the dark. Cells were again washed 3 × in PBS. Images were acquired using a plate reader (Cytation5, Biotek, Winooski, VT, USA).

F-actin fiber length was quantified using Matlab (Mathworks, Natick, MA, USA), with exclusion criteria set to fibers of < 15 μm and > 250 μm length, based on mathematical outlier considerations. The average fiber length for each condition was determined by averaging data from 10 images per well and 8 - 16 wells per treatment condition. The intensity of G-actin immunolabel was quantified in Matlab (Mathworks) by thresholding the image and determining mean intensity per area. The average G-actin expression for each condition was determined by averaging intensity data from 10 images per well and 8–16 wells per treatment condition.

### 2.7. Quantification of Oxidative Stress Using CellROX^®^ Green

Optic nerve head astrocytes were seeded in 8-well chamber slides at a density of 25,000 cells/cm^2^. After the desired treatment, CellROX^®^ Green was added to the wells at a final concentration of 5 μM in complete medium and incubated with the cells for 30 min at 37 °C. Media with CellROX^®^ reagent was removed from the wells by aspiration and cells were rinsed 3 × in PBS. Cells were fixed in 4% PFA for 15 min, then rinsed again 3 × in PBS. Cells were subsequently permeabilized in 0.1% Triton X-100 for 5 min. Nuclei were stained with NucBlue^TM^ Fixed Cell ReadyProbes^TM^ Reagent (Thermo Fisher Scientific, Waltham, MA, USA) at 2 drops per ml of PBS. Cells were rinsed 3 × in PBS. Chamber slides were mounted by placing a coverslip onto the slide using Aqua-Poly/Mount (Polysciences Inc., Warrington, PA, USA). Images were acquired using a Leica SPE confocal microscope (Leica Microsystems Inc., Buffalo Grove, IL, USA).

CellROX^®^ Green fluorescent area was quantified using Fiji software. Briefly, images were converted to 8-bit grayscale and thresholded. The thresholded image was converted into a binary image and an image mask outlining the nuclei created. Analogously, the channel with CellROX^®^ fluorescence was thresholded and the nuclei mask was overlaid. The percent area of nuclear fluorescence was quantified by dividing the area of fluorescence by the total nuclear area. Data are from 10 images per well, and 3–4 separate wells were analyzed per experiment.

### 2.8. Quantification of Oxidative Stress Using Dichlorofluorescein

For quantification of ROS using the dichlorofluorescein method, optic nerve head astrocytes were seeded in 96-well plates at a density of 25,000 cells/cm^2^ and incubated overnight. Media was removed and cells were incubated with 5 μM 6-carboxy-2′, 7′-dichlorodihydrofluorescein diacetate (Carboxy-H_2_DCFDA, Thermo Fisher Scientific, Waltham, MA, USA) in HBSS supplemented with 10 mM HEPES at 37 °C for 30 min. Subsequently, cell supernatant was removed and replaced by complete medium. Following a 30-min incubation, cells were exposed to the desired treatments. After treatments, dichlorofluorescein fluorescence, the product of cleavage of Carboxy-H_2_DCFDA by ROS, was measured at 488 nm excitation/ 520 nm emission using a plate reader (Cytation5, Biotek, Winooski, VT, USA).

### 2.9. Cell Viability Assays

To determine the glioprotective effects of antioxidants on reactive astrocytosis, we conducted 3-(4,5-dimethylthiazol-2-yl)-2,5-diphenyltetrazolium bromide (MTT) uptake and lactate de-hydrogenase (LDH) release assays were performed, as previously described by us in detail [5,6]. In brief, supernatants (50 µL) were collected and LDH assays performed. Cells were incubated with MTT dye for 1.5 h and subsequently lysed in dimethylsulfoxide (DMSO). Data were normalized to the baseline control condition and expressed as fold-change.

### 2.10. Data Analysis and Statistics

All data were analyzed, with the investigator blinded to treatment group. For immunoblotting, all samples were assigned a random number and samples were loaded in random order on acrylamide gels to minimize technical and investigator bias. Data are presented as mean ± standard error of mean (SEM). Open and filled circles represent individual data points. Data were analyzed using paired or unpaired Student’s *t*-test, one-way analysis of variance (ANOVA) or Kruskal–Wallis ANOVA, or Two-Way ANOVA. Differences between groups were subsequently determined using either Tukey’s, Dunn’s, or Sidak’s multiple comparison test, as appropriate. Differences were considered statistically significant at the *p* < 0.05 level.

## 3. Results

### 3.1. Reactive Astrocytosis Causes Cytoskeletal Remodeling and Generation of Increased Cellular Levels of Oxidative Stress

Astrocytes undergoing reactive astrocytosis display a myriad of distinct biochemical and functional phenotypes, including cytoskeletal remodeling and excess generation of cellular oxidative stress. In order to induce a cellular phenotype of reactive astrocytosis, optic nerve head astrocytes were exposed to hyperbaric pressure at 35 mm Hg above ambient pressure for a period of 16 h in a hyperbaric pressure chamber at 37 °C and 5% CO_2_/95% humidity. Control cultures were maintained at ambient pressure.

**Figure 1:**
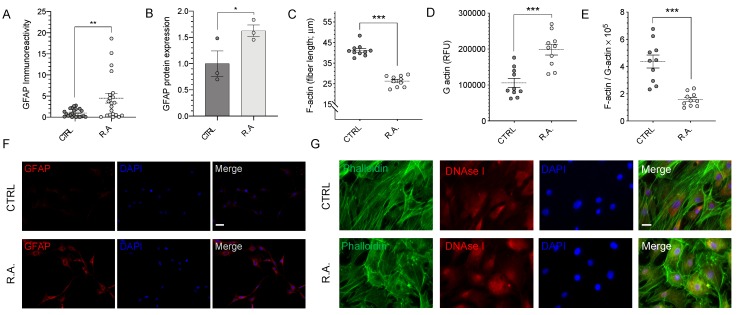
Reactive astrocytosis results in cytoskeletal remodeling. (**A**) anti-glial fibrillary acid protein (GFAP) immunoreactivity was quantified by fluorimetry. A statistically significant increase in GFAP immunoreactivity was observed in activated astrocytes compared to control (*n* = 20–21; *p* < 0.01). (**B**) Quantitative immunoblot analysis revealed a statistically significant increase in GFAP protein expression in activated astrocytes (*n* = 3; *p* < 0.05). (**C**) Quantification of individual F-actin fiber lengths revealed a statistically significant 36% ± 2% decrease in activated optic nerve head astrocytes (*n* = 10; *p* < 0.001). (**D**) A statistically significant 87% ± 12% increase in G-actin immunofluorescence was observed in activated astrocytes (*n* = 10; *p* < 0.001). (**E**) The F-actin to G-actin ratio showed a statistically significant decrease in activated astrocytes (*n* = 10, *p* < 0.001). (**F**) Representative images of GFAP immunocytochemistry. (**G**) Representative images of actin staining. Oligomeric F-actin and monomeric G-actin were visualized using ActinGreen^TM^ ReadyProbes^TM^ Reagent and Alexa Fluor^TM^ 594 conjugated DNAseI, respectively. Data were analyzed by Student’s *t*-test; * *p* < 0.05, ** *p* < 0.01, *** *p* < 0.001. Scale bar: (**F**) 25 μm, (**G**) 10 µm. CTRL = control; R.A. = reactive astrocytosis.

Induction of reactive astrocytosis resulted in significantly elevated GFAP levels (Figure 1A–B,F). Intensity of GFAP immunoreactivity increased 4.52-fold in reactive astrocytosis compared with control optic nerve head astrocytes (*n* = 21–22, *p* < 0.01, Figure 1A). These results were confirmed by quantitative immunoblot for GFAP, which revealed a 1.63-fold increase in expression of GFAP (*n* = 3, *p* < 0.05, Figure 1B).

To determine the effect of reactive astrocytosis on the actin cytoskeleton, optic nerve head astrocytes were labeled with fluorescently-conjugated phalloidin to detect F-actin and DNAse I to detect G-actin. Control astrocytes exhibited long, parallel bundles of actin filaments with modest G-actin expression; activated optic nerve head astrocytes, in contrast, showed significant disorganization of F-actin filaments (Figure 1G). The quantification of F-actin individual fiber length revealed a 36% ± 2% shortening of actin fibers in activated optic nerve head astrocytes (41.2 ± 0.9 µm; *n* = 10) compared with control (26.3 ± 0.8 µm, *n* = 10; *p* < 0.001; Figure 1C). This actin fiber shortening was associated with a concomitant 87% ± 12% increase in the expression levels of monomeric G-actin, as determined by fluorimetry (*n* = 10, *p* < 0.001; Figure 1D, G). In order to test the dependence of the two parameters, the ratio of F-actin fiber length to G-actin expression was calculated, revealing a 68.2% ± 3.6% reduction associated with reactive astrocytosis (*n* = 10, *p* < 0.001; Figure 1E).

**Figure 2:**
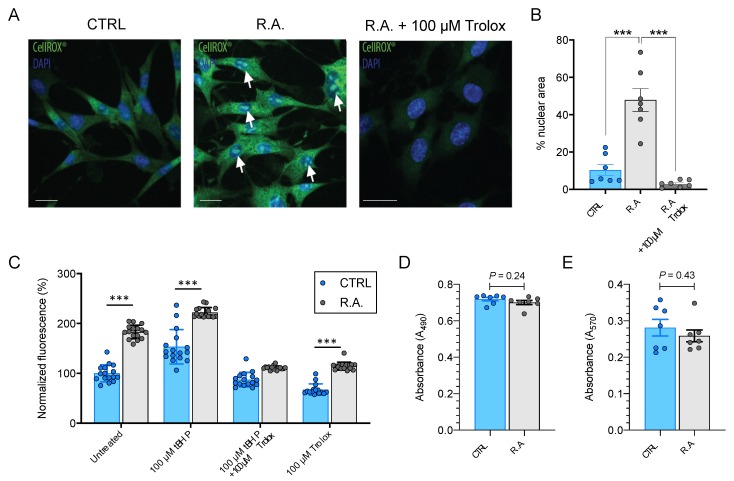
Reactive astrocytosis results in elevated cellular levels of oxidative stress. (**A**) Representative images of CellROX^®^ Green staining for ROS generation. Increased nuclear and cytosolic fluorescence were observed in activated optic nerve head astrocytes compared to control. Pretreatment with 100 µM Trolox for 1 h prior to the induction of reactive astrocytosis prevented this increase in oxidative stress. White arrows point to nuclear fluorescence indicative of ROS generation. (**B**) ROS generation was quantified as percentage of nuclear area covered by CellROX^®^ Green fluorescence (*n* = 7; One-Way ANOVA with Tukey’s multiple comparisons test, *p* < 0.001). Trolox prevented this increase (*n* = 7; *P* < 0.001). (**C**) Dichlorofluorescein fluorescence increased significantly during reactive astrocytosis (*n* = 16, *p* < 0.001). Pretreatment with a sublethal (100 µM) dose of *t*BHP for 1 h prior to and during induction of reactive astrocytosis further increased cellular levels of oxidative stress (*n* = 16, *p* < 0.001). Trolox prevented both the *t*BHP- and reactive-astrocytosis-induced upregulation of oxidative stress (*p* > 0.05). Sixteen individual replicate datapoints are shown, representative of three separate experiments. Data were analyzed by Two-Way ANOVA (*p* < 0.001) with results from Sidak’s multiple comparisons test indicated by asterisks. (**D**) Induction of reactive astrocytosis did not result in a loss of cell viability, as determined by comparing absolute LDH release by measuring A_490_ in control and activated optic nerve head astrocytes (*n* = 7, *p* = 0.24). (**E**) Similarly, there was no statistically significant difference in MTT absorbance (A_570_) between control and activated optic nerve head astrocytes (*n* = 7, *p* = 0.43). Data are shown as mean ± s.e.m. *** *p* < 0.001. CTRL = control; R.A. = reactive astrocytosis. Scale bar = 10 µm.

Cellular levels of oxidative stress were detected using CellROX^®^ Green. Reactive astrocytosis resulted in significant nuclear and cytosolic fluorescence, indicative of elevated cellular levels of oxidative stress that could be prevented by pre-treatment with the prototypic antioxidant, Trolox (Figure 2A). CellROX^®^ Green staining was quantified by estimating the percentage of the total nuclear area covered by fluorescent CellROX^®^ signal (Figure 2B). Reactive astrocytosis increased nuclear fluorescence 4.6 ± 0.6-fold (from 10.4% in control to 47.9% in activated astrocytes; *n* = 7; ANOVA *p* < 0.001; Tukey’s multiple comparisons test, *p* < 0.001), while pre-treatment with Trolox reduced the area of nuclear fluorescence to 28.6 ± 0.1% of baseline (*n* = 7, *p* < 0.001; Figure 2B).

Results from the semi-quantitative CellROX^®^ assay were confirmed by the plate reader-based quantification of dichlorofluorescein labeling. Reactive astrocytosis increased dichlorofluorescein fluorescence intensity by 83% ± 14% (*n* = 16, *p* < 0.001; Figure 2C) compared to control. The exposure of cultures to a sublethal (100 µM) concentration of *t*BHP resulted in a 53% ± 35% increase in fluorescence compared with control untreated optic nerve head astrocytes (*n* = 16, *p* < 0.001; Figure 2C). Reactive astrocytosis further increased fluorescence to 122% ± 10% of control untreated cells (*n* = 3, *p* < 0.001; Figure 2C). Trolox prevented both *t*BHP- and reactive-astrocytosis-induced increases in dichlorofluorescein fluorescence (*p* > 0.05; Figure 2C).

In order to determine whether reactive astrocytosis is associated with a loss of cell viability, LDH and MTT assays were performed. No statistically significant difference in absolute LDH release was identified (*n* = 7, *p* = 0.24; Figure 2D). Similarly, there was no difference in absolute MTT absorbance between control and activated optic nerve head astrocytes (*n* = 7, *p* = 0.43; Figure 2E). These data suggest that reactive astrocytosis at the time point investigated does not cause any detectable reduction in cell viability. 

### 3.2. Different Classes of Antioxidants Elicit Specific Changes in NOX4, SOD2, and Nrf2 Protein Expression

To determine the ability of optic nerve head astrocytes to respond to stimuli by changes in the expression of proteins involved in the endogenous antioxidant response, non-induced optic nerve head astrocyte cultures were exposed to different classes of antioxidants, and the protein expression levels of NOX4, SOD2 and Nrf2 were quantified by immunoblotting.

Specifically, astrocytes were treated with 0.005% Mn-TM-2-PyP or PBS vehicle, 50 μM resveratrol or 0.1% DMSO vehicle, 100 µM Trolox or 0.1% ethanol vehicle, or 5 µM xanthohumol or 0.01% DMSO vehicle for 24 h. Concentrations were determined based on previous studies [5,6,19], the published literature [23,29,30] and cytotoxicity studies in optic nerve head astrocytes (Figure S1). Quantitative immunoblotting was performed to determine NOX4, SOD2, and Nrf2 protein expression.

**Figure 3:**
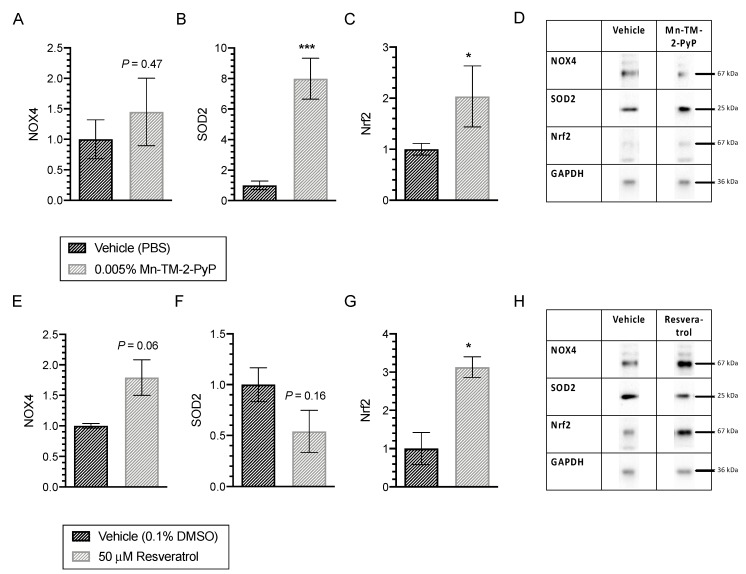
Differential effects of Mn-TM-2-PyP and resveratrol on expression levels of Nrf2, SOD2, and NOX4 in control optic nerve head astrocytes. (**A–C**) Mn-TM-2-PyP elicited no significant effect on NOX4 expression (*n* = 3–6; *p* = 0.47); Figure 3A), but resulted in a statistically significant 8.0-fold increase in SOD2 (*n* = 3–6; *p* < 0.001) and a 2.0-fold increase in Nrf2 (*n* = 3–6; *p* < 0.05). (**D**) Representative examples from quantitative immunoblotting are shown. (**E–G**) Treatment with resveratrol increased NOX4 protein expression 1.8-fold, but this effect did not reach statistical significance (*n* = 3, *p* = 0.06). No significant effect on SOD2 expression was identified (*n* = 3, *p* = 0.16; Figure 3E). In contrast, Nrf2 expression was elevated 3.1-fold (*n* = 3, *p* < 0.05). Data are presented as mean ± SEM. (h) Representative examples from quantitative immunoblotting are shown. * *p* < 0.05, *** *p* < 0.001.

Exposure to Mn-TM-2-PyP had no significant effect on NOX4 expression (*n* = 3–6; *p* = 0.47; Figure 3A), but resulted in a statistically significant 8.0-fold increase in SOD2 expression (*n* = 3–6; *p* < 0.001; Figure 3B) and a statistically significant 2.0-fold increase in Nrf2 expression (*n* = 3–6; *p* < 0.05; Figure 3C).

Treatment with resveratrol increased NOX4 protein expression 1.8-fold, but this effect did not reach statistical significance (*n* = 3, *p* = 0.06; Figure 3D). While resveratrol had no effect on SOD2 expression (*n* = 3, *p* = 0.16; Figure 3E), Nrf2 expression was elevated 3.1-fold (*n* = 3, *p* < 0.05; Figure 3E).

Trolox and xanthohumol did not exert any significant changes on expression of Nrf2, SOD2 or NOX4 (Table 1).

In a preliminary experiment, we also quantified catalase expression given the significant increase in SOD2 expression after exposure to Mn-TM-2-PyP. Treatment with Mn-TM-2-PyP resulted in a trend toward increased catalase expression (1.00 ± 0.18 vs. 2.53 ± 0.82, *n* = 3, *p* = 0.14). Resveratrol (1.00 ± 0.43 vs. 0.97 ± 0.72, *n* = 3, *p* = 0.97), Trolox (1.00 ± 0.44 vs. 1.11 ± 0.45, *n* = 3, *p* = 0.87) and xanthohumol (1.00 ± 0.43 vs. 1.15 ± 0.26, *n* = 3, *p* = 0.78) had no effect on catalase expression (Figure S2A–H).

### 3.3. Different Classes of Antioxidants Exert Varying Degrees of Glioprotection Against Reactive Astrocytosis- and tBHP- Induced Oxidative Stress

To assess the glioprotective effects of these antioxidants, cell viability of activated and control optic nerve astrocytes in response to increasing doses of exogenously-applied oxidative stress was quantified. MTT and LDH assays were employed as surrogate markers for cell viability.

**Figure 4:**
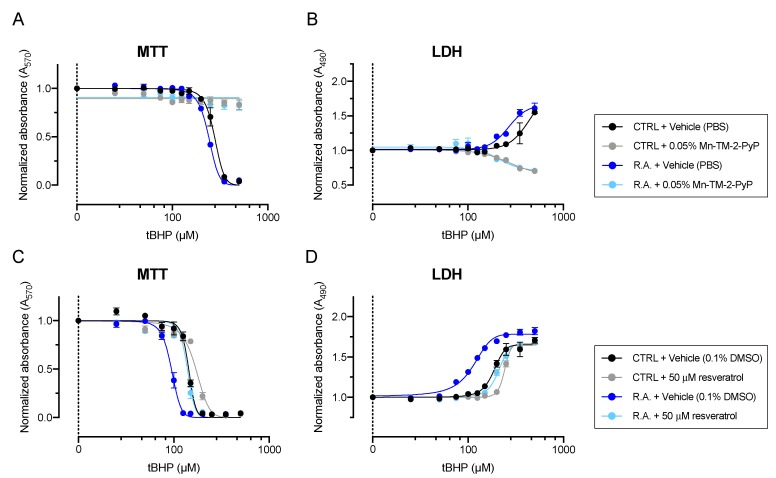
Glioprotective effects of Mn-TM-2-PyP and resveratrol against reactive astrocytosis- and *t*BHP-induced oxidative stress. (**A**) Pretreatment of optic nerve head astrocytes with Mn-TM-2-PyP resulted in complete protection against both reactive astrocytosis- and *t*BHP-induced oxidative stress, as determined by MTT assay. (**B**) Similarly, lactate dehydrogenase (LDH) assay revealed potent glioprotection by Mn-TM-2-PyP. (**C**) Resveratrol exerted strong glioprotective effects that resulted in a statistically significant shift in the IC_50_ value for *t*BHP. (**D**) Similar shifts were observed in the *t*BHP dose-response curve in the LDH assay. Data are shown as mean ± s.e.m. from three (Mn-TM-2-PyP) or four (resveratrol) separate experiments, with eight technical replicates per experiment.

Increased levels of cellular oxidative stress in activated optic nerve head astrocytes were reflected by a sensitization to exogenously applied oxidative stress insult, resulting in a left-ward shift of the dose-response curve to *t*BHP (Figure 4).

Mn-TM-2-PyP resulted in potent glioprotection that completely prevented loss of cell viability in both control and activated astrocytes and no loss of cell viability could be detected in Mn-TM-2-PyP treated astrocytes by MTT (*n* = 3, Figure 4A) and LDH release (*n* = 3, Figure 4B) assays at *t*BHP concentrations up to 500 μM.

Similarly, resveratrol exerted strong glioprotective effects that resulted in a statistically significant shift in the IC_50_ value for *t*BHP from 141 ± 2.5 µM to 175 ± 2.4 µM in control astrocytes (*n* = 4, *p* < 0.001; Figure 4C) and 94.3 ± 2.4 µM to 141 ± 1.5 µM in activated astrocytes (*n* = 4, *p* < 0.001; Figure 4C). These findings were confirmed in the LDH assay, which revealed shifts in the EC_50_ values from 196 ± 4.1 µM to 241 ± 0.9 µM in control astrocytes (*n* = 4–5, *p* < 0.001; Figure 4D) and from 105 ± 3.2 µM to 208 ± 6.5 µM in activated astrocytes (*n* = 4–5, *p* < 0.001; Figure 4D).

Glioprotection by xanthohumol was modest, shifting the IC_50_ for *t*BHP in the MTT assay by 15.9 µM in control astrocytes and 14.5 µM in activated astrocytes (Figure S3A, Table 2). Similarly, the LDH assay revealed shifts of 25.5 µM and 32.9 µM (Figure S3B, Table 2), respectively.

Treatment with 100 μM Trolox caused a right-shift of both the MTT and LDH dose-response curves to *t*BHP that was proportional between control and activated optic nerve head astrocytes (Figure S3C,D, Table 2).

## 4. Discussion

Astrocytes not only sustain neuronal function by providing trophic and metabolic support, but are important for cellular communication. One way for astrocytes to respond to a variety of stimuli is to undergo a process of activation, often referred to as reactive astrocytosis (for review, see [31]). Reactive astrocytes exhibit a phenotype of increased motility and proliferation associated with strong GFAP expression [32].

In the present study, we used GFAP expression as surrogate marker to confirm the induction of reactive astrocytosis in primary rat optic nerve head astrocytes (Figure 1). Increased GFAP expression following the induction of reactive astrocytosis by hyperbaric pressure was confirmed by immunocytochemistry (Figure 1A,F) and immunoblotting (Figure 1B). Several GFAP variants exist that exhibit distinct subcellular mRNA expression [33] and regulation of the intermediate filaments [34]. One study investigated GFAP isoforms in a mouse model for Alzheimer’s Disease but did not identify any association with aging or reactive astrocytosis [35]. If or to what extent differential isoform expression underlies pathological changes or contributes to reactive astrocytosis in optic nerve head astrocytes remains unknown. Therefore, only overall GFAP expression was quantified herein. Future studies will address whether reactive astrocytosis in optic nerve head astrocytes is associated with differential changes in GFAP isoforms.

In the glaucomatous optic nerve head, reactive astrocytes undergo a characteristic remodeling of the actin cytoskeleton, contributing to optic nerve head cupping [36]. Cupping is an important clinical criterion for diagnosing and assessing the progression of glaucoma [37]. Here, reactive astrocytosis resulted in the shortening of F-actin fibers (Figure 1C) and increased G-actin fluorescence (Figure 1D), suggestive of a possible depolymerization of actin. These findings manifested as a noticeable disorganization of actin stress fibers. In control astrocytes, F-actin showed very orderly and linear parallel bundles, while it appeared very disorderly and in a more perpendicular orientation to neighboring bundles in activated optic nerve head astrocytes (Figure 1G). The F-to-G actin ratio (F-actin fiber length over G-actin fluorescence) significantly decreased during reactive astrocytosis (Figure 1E). Altogether, these data are consistent with in vitro studies using 3D culture models [38], in vivo models using an acute controlled elevation of intraocular pressure (CEI) [39], and with the generally accepted notion that mechanical forces exerted on the human optic nerve head cause disruption of the actin cytoskeleton (reviewed in [40]).

The induction of reactive astrocytosis in cultured optic nerve head astrocytes resulted in the generation oxidative stress as quantified using two different approaches. CellROX^TM^ Green becomes highly fluorescent upon oxidation by ROS and subsequent binding to DNA, resulting in strong nuclear and mitochondrial fluorescence (Figure 2A). Nuclear fluorescence was quantified by thresholding images and determining the fractional fluorescent nuclear area (Figure 2B). These findings were confirmed by the quantification of dichlorofluorescein fluorescence using plate-reader-based detection. Reactive astrocytosis resulted in a significant increase in dichlorofluorescein fluorescence. Combining a sublethal concentration of chemically induced oxidative stress by 100 µM *t*BHP with the induction of reactive astrocytosis resulted in a further proportional increase in fluorescence, while the prototypic ROS scavenger, Trolox, was able to prevent any increases (Figure 2C). Non-specific probes for ROS detection, such as dichlorofluorescein, can be oxidized by both peroxides and other ROS species. As the present study did not quantify the amount of cellular peroxide directly, it is impossible to estimate the contribution of peroxides to dichlorofluorescein fluorescence. However, the cell permeable dye, CellROX^®^ Green, primarily detects radical oxygen and radical hydroxyl species [41]. These data suggest that reactive astrocytosis in vitro is associated with a gradual generation of ROS. Notably, reactive astrocytosis was not associated with a loss of cell viability, as determined by LDH and MTT assays.

One limitation of this study is that only one timepoint (16 h induction of reactive astrocytosis) was investigated. However, this timepoint was considered optimal for the subsequent assessment of antioxidant effects for two reasons: 1. astrocyte cultures could be maintained in complete, serum-containing media, while at the same time controlling for confounding effects of proliferation during the induction period; and 2. the induction of reactive astrocytosis was not associated with detectable loss of cell viability. The reactive astrocytosis-associated generation of elevated levels of oxidative stress is in accordance with a rigorous body of work that includes clinical studies and network analysis of human glaucomatous optic nerve head astrocytes [42], as well as in vivo studies [43,44].

Treatment with different classes of antioxidants resulted in differential effects on Nrf2, SOD2, and NOX4 proteins that were concurrent with their glioprotective properties against reactive astrocytosis- and oxidative stress-induced oxidative stress. These proteins were selected based on previously reported mechanisms of action of antioxidants involving Nrf2 and SOD2 [11,15,20,45]. NOX4 is one of the major isoforms of NADPH oxidases (reviewed in [46]). Expressed highly in brain astrocytes [47], NOX4 has been associated with subarachnoid hemorrhage-induced oxidative stress [48]. Furthermore, NOX4 is upregulated in response to transforming growth factor β1 signaling in numerous cell types [49,50,51,52]. Given the effects of Mn-TM-2-PyP on SOD2 expression, we also quantified catalase expression after treatment with antioxidants in control optic nerve head astrocytes. Although not statistically significant, we observed a trend of increased catalase expression after treatment with Mn-TM-2-PyP, likely as a result of increased hydrogen peroxide levels generated from SOD2 activity.

Mn-TM-2-PyP exhibited the most potent glioprotective effects against reactive astrocytosis-induced oxidative stress, associated with a potent upregulation of Nrf2 and SOD2 expression. Mn-TM-2-PyP has previously been shown to be mitochondrially targeted, which may also contribute to its strong glioprotective effect [12]. Further studies must investigate whether this compound is entering the mitochondria of reactive optic nerve head astrocytes to evaluate its potential therapeutic effects. Manganese porphyrins have an established preclinical safety profile [24] and are currently used as an adjunctive therapy concurrent with radiation therapy (clinical trials #NCT03386500 and # NCT02655601). Their solubility and stability in aqueous buffers and formulations make manganese porphyrins, including Mn-TM-2-PyP, excellent candidates for a rapid translation to the clinic.

Resveratrol has been proposed to exert its antioxidant effects through a plethora of distinct mechanisms that include modulation of the endogenous antioxidant response element via Nrf2 [22,53], direct effects on Ca_V_ and other types of calcium channels [29], as well as direct antioxidant and antiperoxidant effects [54]. Notably, resveratrol has also been shown to inhibit histone deacetylases, which may alter the gene transcription of endogenous antioxidant enzymes [55]. Recently, an elegant study in rat optic nerve head astrocytes found that optic nerve head astrocytes respond to oxidative stress insult by proteolytic cleavage of Tau by caspases and the subsequent formation of neurofibrillary tangles, analogous to neurons [30]. Furthermore, the study showed that treatment with 100 µM resveratrol was able to prevent Tau cleavage [30]. These data complement the findings reported herein that resveratrol is highly glioprotective, likely due to multiple intracellular mechanisms of action.

Xanthohumol exerts antioxidative effects by increasing Nrf2 translocation to the nucleus by facilitating the dissociation of Keap1 from Nrf2 [56]. Surprisingly, no effect on Nrf2 or any of the phase II antioxidant enzymes tested was identified in this study (Table 1). Xanthohumol belongs to the groups of flavonoids and, therefore, possesses direct ROS scavenging activity, which may be responsible for the observed modest glioprotective effects in optic nerve head astrocytes [45]. Additionally, it may be beneficial to investigate nuclear versus cytoplasmic Nrf2 expression with Xanthohumol to determine if it is increasing Nrf2 translocation in this cell type.

Overall, the expression of SOD2 and Nrf2 largely corresponded to the antioxidant effects of the compounds. Xanthohumol’s inability to modulate the endogenous antioxidant proteins at a single time point tested corresponded to its minimal glioprotective effect observed in this study. Typically, the endogenous antioxidant system responds within a few hours to environmental changes. While resveratrol and Mn-TM-2-PyP both led to similar increases in Nrf2 expression, the ability of Mn-TM-2-PyP to sustain the significant upregulation of SOD2 at 24 h after Mn-TM-2-PyP treatment may explain its potent glioprotective and cytoprotective effects (present study; [19]).

Previous studies in human optic nerve head astrocytes have investigated Nrf2 expression primarily at the gene expression level in response to 4-hydroxynonenal treatment [57] or reported the response of heat shock protein (Hsp) α_B_-crystallin and Hsp27, fibronectin and connective tissue growth factor [58]. The present study is, to our knowledge, the first to describe the differential effects of different classes of antioxidants on NOX4, SOD2 and Nrf2 expression levels in optic nerve head astrocytes and to test their effects in a disease-relevant state of activation. In addition, the present study is the first to describe the expression of NOX4 in optic nerve head astrocytes. Future studies will address the expression of other isoforms of pro-oxidant NOX enzymes in optic nerve head astrocytes and their potential role in reactive astrocytosis.

## 5. Conclusions

Our results provide preliminary feasibility data supporting the preclinical development of antioxidants as novel therapeutics targeting the glaucomatous optic nerve head. By targeting the site of some of the pathological changes, glioprotective agents may be developed into efficacious therapies that can ultimately slow or prevent some of the deleterious pathologies associated with optic neuropathies.

## Figures and Tables

**Table 1:** Lack of effect of xanthohumol and Trolox on Nrf2, SOD2, and NOX4 expression in primary optic nerve head astrocytes.

Antioxidant	Condition	*Nrf2*	*SOD2*	*NOX4*
Trolox	Vehicle (0.1% ethanol)	1.00 ± 0.22	1.00 ± 0.35	1.00 ± 0.04
	Trolox (100 µM)	1.62 ± 0.02	1.26 ± 0.51	1.79 ± 0.29
		*n* = 3, *p* = 0.12	*n* = 3, *p* = 0.70	*n* = 3, *p* = 0.06
Xanthohumol	Vehicle (0.01% DMSO)	1.00 ± 0.42	1.00 ± 0.17	1.00 ± 0.22
	Xanthohumol (2.5 µM)	0.98 ± 0.29	1.07 ± 0.61	0.73 ± 0.32
		*n* = 3, *p* = 0.97	*n* = 3, *p* = 0.91	*n* = 3, *p* = 0.52

**Table 2:** Trolox and xanthohumol are glioprotective against reactive astrocytosis- and chemically induced oxidative stress. (n.d. = not determined).

Antioxidant	Condition	Control		Reactive Astrocytosis	
		MTT IC_50_ (tBHP)	LDH EC_50_ (tBHP)	MTT IC_50_ (tBHP)	LDH EC_50_ (tBHP)
**Trolox**	Vehicle (0.1% ethanol)	73.0	126.7	50.0	93.1
	Trolox (100 µM)	135.2	n.d.	99.3	210.3
Xanthohumol	Vehicle (0.01% DMSO)	143.2	185.4	94.4	113.0
	Xanthohumol (2.5 µM)	159.1	210.9	108.9	145.9

## Supplementary Materials

The following are available online at https://www.mdpi.com/2076-3921/9/4/324/s1, Figure S1: Cytotoxicity of antioxidants on optic nerve head astrocytes, Figure S2: Effects of antioxidants on expression levels of CAT in control optic nerve head astrocytes, Figure S3: Glioprotective effects of Trolox and xanthohumol against oxidative stress.
