# Supplementary material for: Differential Activation of Glioprotective Intracellular Signaling Pathways in Primary Optic Nerve Head Astrocytes after Treatment with Different Classes of Antioxidants
Source: Antioxidants (Basel). 2020 Apr 16;9(4):324. doi: 10.3390/antiox9040324 (PMC7222350; doi:10.3390/antiox9040324)
Supplement: Supplementary file 1 [file antioxidants-09-00324-s001.pdf]

Supplementary Materials

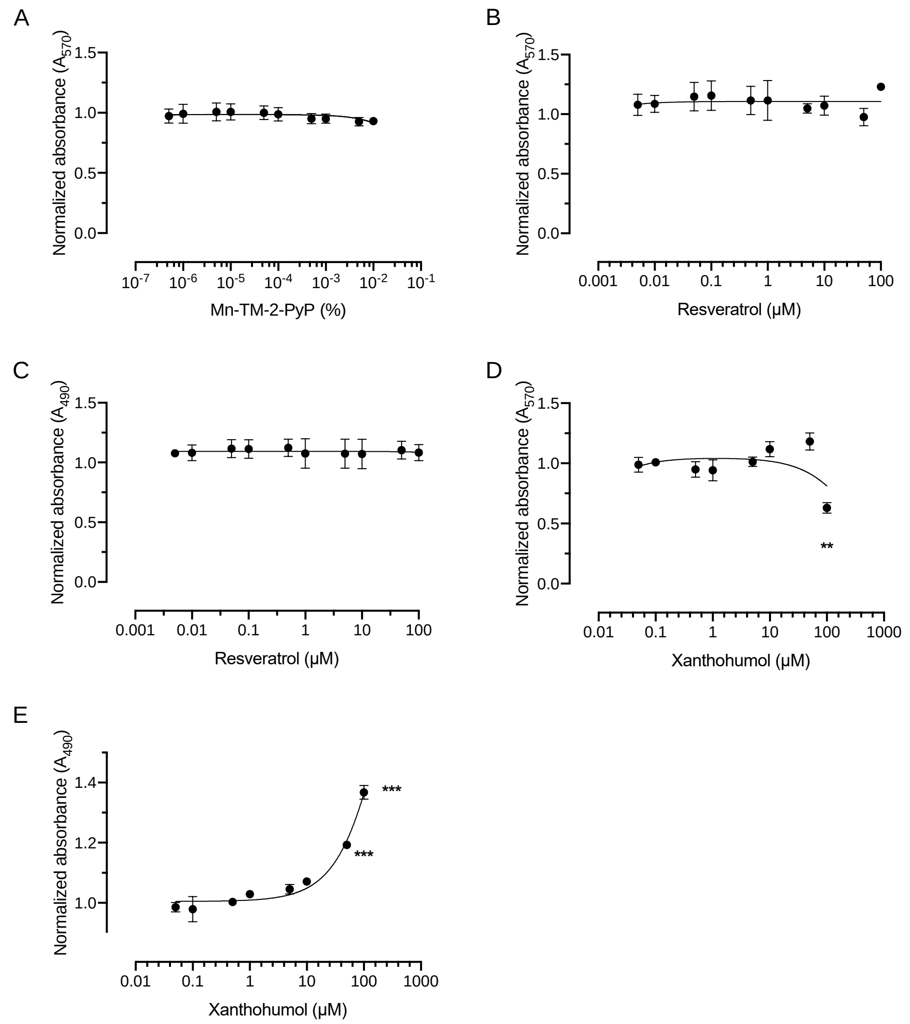

Figure S1. Dose-response cytotoxicity curves of antioxidants on optic nerve head astrocytes. (**a**) Mn-TM-2-PyP did not exert any significant cytotoxicity at concentrations up to 0.01% (w/v) in the MTT assay. Mn-TM-2-PyP was diluted in PBS. (**b**/**c**) Resveratrol did not exert any significant cytotoxicity at concentrations up to 100 μM when dissolved in 0.1% DMSO. (**d**) Xanthohumol resulted in a statistically significant loss of cell viability as determined by the MTT assay at a concentration of 100 μM (n = 3, $P < 0.01$), when dissolved in 0.01% DMSO. (**e**) Similarly, xanthohumol resulted in increased LDH release at concentrations of 50 μM and 100 μM (n = 3, $P < 0.001$).

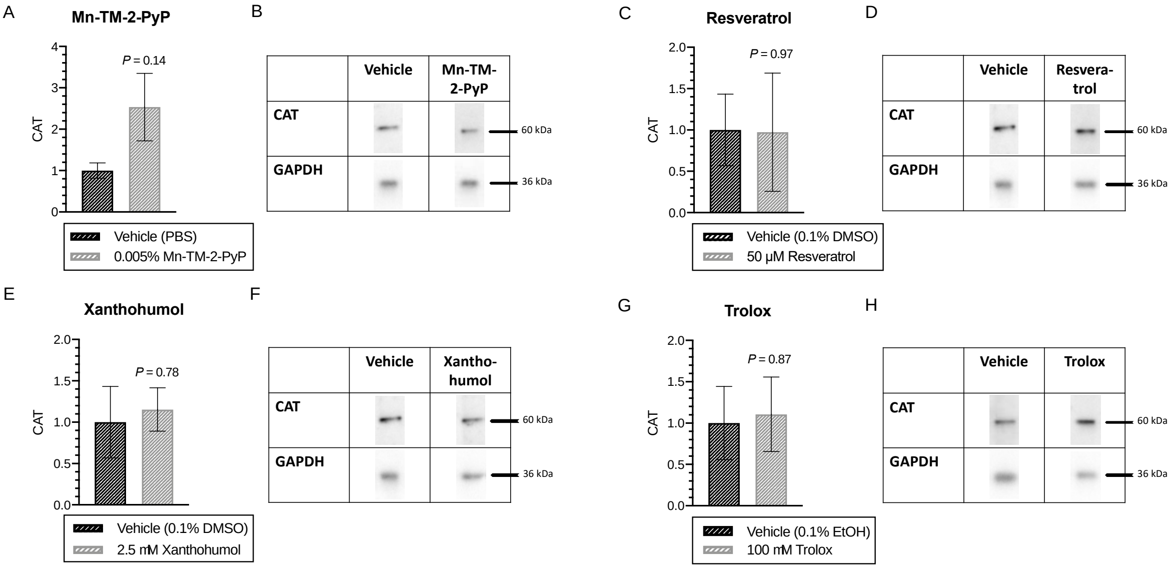

Figure S2. Effects of antioxidants on expression levels of CAT in control optic nerve head astrocytes. (**a**) Treatment with Mn-TM-2-PyP resulted in a trend toward increased catalase expression (1.00 ± 0.18 *vs*. 2.53 ± 0.82, n = 3, *P* = 0.14). (**b**) Representative examples from quantitative immunoblotting are shown. (**c-g**) Resveratrol (1.00 ± 0.43 *vs*. 0.97 ± 0.72, n = 3, *P* = 0.97), Trolox (1.00 ± 0.44 *vs*. 1.11 ± 0.45, n = 3, *P* = 0.87) and xanthohumol (1.00 ± 0.43 *vs*. 1.15 ± 0.26, n = 3, *P* = 0.78) had no effect on catalase expression. Data are shown as mean ± s.e.m.

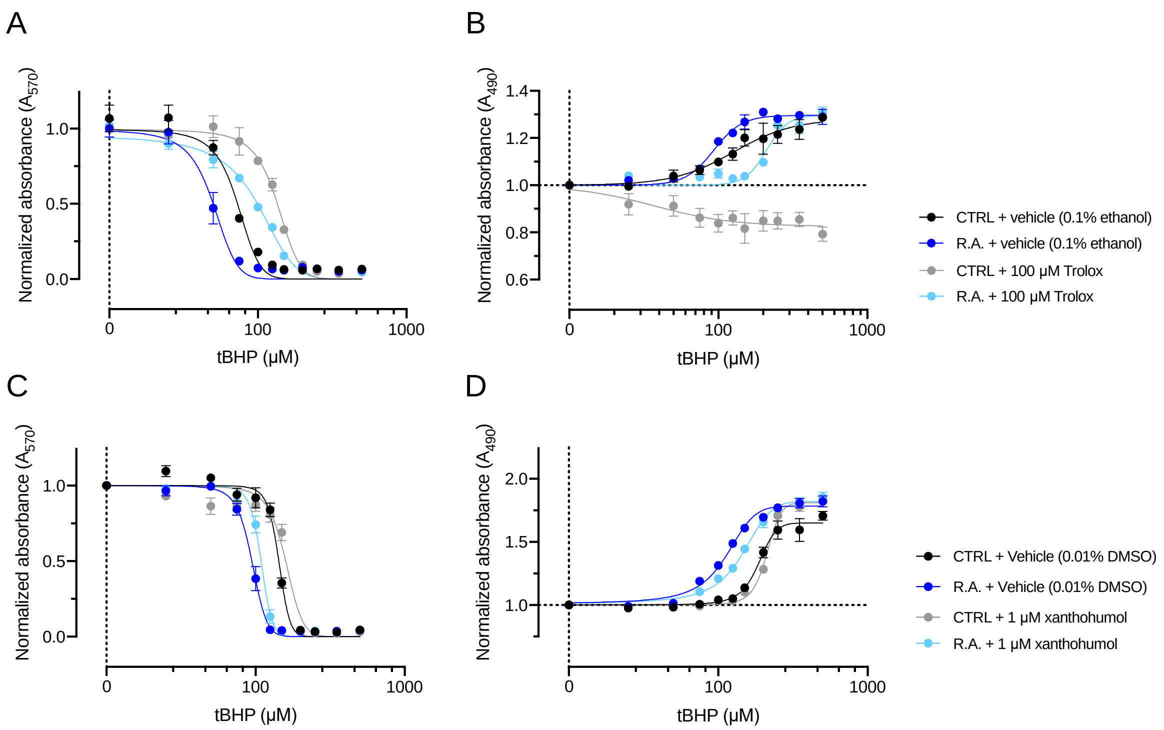

Figure S3. Glioprotective effects of Trolox and xanthohumol against reactive astrocytosis- and *t*BHP-induced oxidative stress. (**a**) Pretreatment of optic nerve head astrocytes with Trolox (100 µM) resulted in potent glioprotective effects against both reactive astrocytosis- and *t*BHP-induced oxidative stress, as determined by MTT absorbance. (**b**) Similarly, LDH assay revealed potent glioprotection by Trolox. (**c**) Xanthohumol exerted modest glioprotective effects that resulted in a statistically significant shift in the IC_{50} value for *t*BHP. (**d**) Similar shifts were observed in the *t*BHP dose-response curve in the LDH assay. Data are show n as mean ± s.e.m.
